# Supplementary material for: The role of cofeeding arthropods in the transmission of Rickettsia felis
Source: PLoS Negl Trop Dis. 2022 Jun 27;16(6):e0010576. doi: 10.1371/journal.pntd.0010576 (PMC9269922; doi:10.1371/journal.pntd.0010576)
Supplement: Attachment — Submitted filename: Response Letter PNTD-D-22-00511_6-7-22 final.docx [file pntd.0010576.s001.docx]

Ref. No.:  PNTD-D-22-00511

Reviewers' Comments to Authors:

Reviewer #1:

Overall, the manuscript is well and clearly written.  The introduction of *R. felis* transmission by co-feeding with potential vectors/hosts will initiate further investigations to clarify the diversity and breath of vectors of this unusual rickettsial pathogen.

***Response:*** Thank you for the reviewer’s positive comments.

Reviewer #2:

The nidality of arthropod-borne rickettsial disease systems are fascinating and, at times, can boggle the mind.  The *Rickettsia felis* system is particularly interesting with publications constantly popping up, reporting its presence in all kinds of arthropods.  This study takes it one step further and moves into the question ‘how does this rickettsial pathogen get around so freely?’  I really enjoyed reading this paper and commend the research team for thinking through the feeding trials in such a way that produced some pretty clear results.  In summary, with some minor revisions and clarifications, I feel this paper will be ready - and will help to fill in some gaps in our knowledge regarding how *R. felis* moves between different hosts.

***Response:*** Thank you for the reviewer’s positive comments. In the text below in minor comments we provide the response to the comments raised by the reviewer.

Reviewer #3:

The project in this manuscript used several bioassays to explore the role of cofeeding arthropods in the transmission of *Rickettsia felis*, an important emerging rickettsial pathogen. This reviewer enjoyed reading this manuscript which is well-written. The methods related to this project are described in the details, and the conclusion of this manuscript is well supported by the performed assays. The authors are encouraged to address the below comments:

1. The authors may consider including the keyword- the organism (*Rickettsia felis*) as part of the manuscript title.

***Response:*** Thank you for the reviewer’s positive comments. The suggested change was made to the title.

2. *R. felis* has been identified in multiple vectors, including different species of ticks and mosquitoes. The authors shall provide the rationale regarding the choice of the tick and mosquito species used in this project. In addition, please discuss if the conclusions drawn by the use of the tick and mosquito species in this work will apply well to other tick species and mosquito species.

***Response:*** In this current study, we suggest a model for inter-class and inter-order transmission of *R. felis* in hematophagous arthropods. The species were chosen based on our laboratory studies and availability of *Anopheles* species. Outside of the two species examined, we suspect that this route of transmission would be applicable to the spread of *R. felis* in other species of ticks and mosquitoes. We have added two sentences to the last paragraph of the discussion to clarify these points. Specific text added:

“The arthropod species examined in this study were selected based on laboratory studies and species availability. The susceptibility of other species of ticks and mosquitoes is not known, but the diversity of arthropods associated with *R. felis* suggests widespread susceptibility of other species to *R. felis*.”

3. The starting copy number of *R. felis* is very high ((3 × 10~10 rickettsiae/700 μl). Then, the detected rickettsial copies in ticks and mosquitoes are much lower, particularly in mosquitoes. Could the *R. felis* positivity in mosquitoes be due to the carry-over of *R. felis* from the original organism? The qPCR, not the culture, was used to determine the copy number of *R. felis*, not the viability of the pathogen.

***Response:*** The mosquitoes and ticks were collected at 24 h post-exposure for the first time point, so *R. felis* should be ingested. Also, all samples were surface sterilized before gDNA extraction, with appropriate environmental controls. Thus, the *R. felis* positive should come directly from the mosquitoes and ticks. Viability and subsequent dissemination in recipient arthropods does require further study.

4. Regarding the transmission of *R. felis* from fleas to cofeeding mosquitoes, lines 48-50 reads: Similar to ticks, mosquitoes were able to acquire *R. felis* while cofeeding with infected cat fleas on a vertebrate host, with infection persisting in the mosquito for up to 3 dpe. In this experiment, the *R. felis* identified in mosquitoes can possibly acquire the organism from cat flea and/or the host? Or, more likely from the host?

***Response:*** In this experiment we report that mosquitoes acquired *R. felis* via cofeeding with infected cat fleas. We surface sterilize the samples prior to gDNA extraction. Given that not all recipients arthropods were positive for *R. felis*, we suspect that transmission via the host, not contact between the vectors, accounts for the positive recipients.

Minor comments:

Reviewer #1:

1. Title: Would add to or replace emerging rickettsial pathogen the name of the pathogen *Rickettsia felis*.

***Response:*** Thank you, the suggested change has been made in title.

2. Abstract: Lines 44 and 48: Would suggest that you include the name of the vertebrate host e.g. rat.

***Response:*** Thank you, rat was added instead of a vertebrate host. (Line 43, and 48)

3. Introduction:
   3.1. Line 70: remove the word “by”.

***Response:*** Thank you, the suggested change made in the text. (Line 71)

3.2 Line 78: suggest removing the word “vectors” and making the last word plural, i.e. arthropods.

***Response:*** Thank you, the suggested change made in the text. (Line 78)

3.3 Line 80: suggest replacing “vectors” with arthropods.

***Response:*** Thank you, the suggested change made in the text. (Line 80)

4. Materials and Methods:
   4.1. Line 106: scapularis in italics i.e. scapularis-derived

***Response:*** Thank you, the suggested change made in the text. (Line 106)

4.2. Line 116: suggest defining qPCR as quantitative real-time PCR

***Response:*** Thank you, the suggested change made in the text. (Line 115)

4.3. Line 138: what was the portion of the donor cat fleas e.g. 10 or 10%?

***Response:*** The portion of the donor cat fleas is the number of *R. felis*-infected donor cat fleas that we collected at 24 h post-exposure, which ranged from 25-40% of the total number of *R. felis*-infected donor cat fleas added to the cofeeding capsules. Because the numbers varied between the replicates, we reported the total N in the results.

4.4. Line 140: a portion, maybe this is written in the results?

***Response:*** We presented a number *D. variabilis* nymphs in Table 1, Column 3 shows % Positive (n). n is the number of nymphs that we collected for gDNA extraction.

5. Results:

5.1 Line 195: add Sprague Dawley or SD rat after vertebrate host.

***Response:*** Thank you, the suggested change was made in the text. (Line 196)

5.2 Line 200: add number of positive adult ticks/number evaluated

***Response:*** We added a number of positive adult ticks/number evaluated in the results. (Line 201-203)

6. Table 1: Define ND

***Response:*** Defined ND as not detected - has been added to Table 1.

7. Discussion:
   Line 252: Rickettsia senegalensis should be written as Candidatus Rickettsia senegalensis.

***Response:*** Thank you, the suggested change was made in the text. (Line 246)

Reviewer #2:

1. Line 43: transmit should be past-tense – transmitted

***Response:*** Thank you, the suggested change was made in the text. (Line 42)

2. Line 45: There is no comma needed between observed and with.

***Response:*** Thank you, the suggested change was made in the text. (Line 45)

3. Line 48: No comma needed between host and with

***Response:*** Thank you, the suggested change was made in the text. (Line 48)

4. Line 53: The way this is written it seems to say that R felis is the common cat flea - take out the comma after felis

***Response:*** Thank you, the suggested change was made in the text. (Line 54)

5. Line 60: Can take out 'other arthropods including' as ADT and mosquitoes were the only ones tested

***Response:*** Thank you, the suggested change was made in the text. (Line 61)

6. Line 65: ‘with’ normally does not follow a semi-colon (;) so you can remove it.  Might be better to phrase it: 'rash followed by complications involving neurological systems (1).

***Response:*** Thank you, the suggested change was made in the text. (Line 66-67)

7. Line 68: Better to change the tense: ‘have been increasing’ to ‘have increased’

***Response:*** Thank you, the suggested change was made in the text. (Line 68)

8. Line 106: scapularis needs to be italicized

***Response:*** Thank you, the suggested change was made in the text. (Line 106)

9. Lines 193-209: The data here is good but I struggled while I read with the question: how do they differentiate between live *R. felis* and DNA from dead *R. felis* in *D. variabilis* nymphs?  I realize the qPCR measures that - but I feel it could be stated more clearly in this section of the results so the reader isn't wondering what kind of DNA is being detected.  For example, this aspect was taken care of in the mosquito section by presenting the linear changes in rickettsial concentration over the different time points.

***Response:*** We agree with the reviewer that we cannot differentiate between live and dead *R. felis* in *D. variabilis* by qPCR. However, in this experiment we only expected to determine the potential of *R. felis* transmission from the donor cat fleas to *D. variabilis* nymphs. Although we did not enumerate rickettsial growth during the process of tick feeding and molting, we can only speculate if the DNA was still present, it represents viable rickettsiae.

10. Line 292: No need for semi-colon between ‘felis’ and ‘with’

***Response:*** Thank you, the suggested change was made in the text. (Line 286)

11. Lines 301-321: There are several instances in this paragraph where there is a need to clarify ideas. Please re-work the whole paragraph so the ideas read more smoothly.

***Response:*** We have revised the sentences and structure of the paragraph for clarity.

12. Lines 302-305: There are quite a few comma phrases here in this four line sentence.  I would suggest making two sentences out of it so the ideas flow better.

***Response:*** We have modified the text for clarity and flow. (Line 303-305)

13. Lines 308: no comma needed after mosquitoes.  Could even change it to ‘which, in the lab, are able...’

***Response:*** Thank you, the suggested change was made in the text. (Line 302)

14. Lines 309-311: sentence is a bit awkward - please smooth - two very different thoughts connected by an 'and'

***Response:*** We have modified the text for clarity and flow. (Line 309-311)

15. Lines 311-315: Again, these are confusing sentences.  Please work to smooth out the storyline as ideas feel like they're jumping around.

***Response:*** We have modified the text for clarity and flow – specifically removed disjointed text.

16. Lines 326-329: Key to highlight here is the need for transmission studies.  I realize it is mentioned in the last sentence but it needs to be brought out more because only one aspect of competency has been demonstrated here (acquisition by the non-flea host) – transmission by the non-flea host still needs to be demonstrated.

***Response:*** We appreciate the comment and hope by concluding with the stated need to examine vector competency of other arthropods we present the needs in research to the readers.
